# Evolving Referral Patterns and Management Following Implementation of a Multidisciplinary Fibroid Center: A Retrospective Cohort Study

**DOI:** 10.3390/jcm13133632

**Published:** 2024-06-21

**Authors:** Kelsey Musselman, Kristen Pepin, Nicole A. Lamparello, Yelena Havryliuk, Marc Schiffman, Tamatha Fenster, Ja Hyun Shin

**Affiliations:** 1 Department of Gynecology and Obstetrics, Johns Hopkins Hospital, Baltimore, MD 21287, USA; 2 Department of Obstetrics and Gynecology, Weill Cornell Medicine, New York, NY 10021, USA; kjp9013@med.cornell.edu (K.P.); yeh9001@med.cornell.edu (Y.H.); taf2011@med.cornell.edu (T.F.); jas7013@med.cornell.edu (J.H.S.); 3 Department of Vascular and Interventional Radiology, Weill Cornell Medicine, New York, NY 10021, USA; nil9053@med.cornell.edu (N.A.L.); mas9252@med.cornell.edu (M.S.)

**Keywords:** fibroids, interdisciplinary, fibroid program, collaborative care, uterine artery embolization

## Abstract

**Background/Objectives**: Our objective was to evaluate changes in the management of symptomatic fibroids after establishing a multidisciplinary fibroid center with minimally invasive gynecologic surgery (MIGS) and interventional radiology (IR). **Methods**: A retrospective cohort study was conducted at the fibroid center created in September 2020. Patients were offered same-day consults with both MIGS and IR providers. Data were collected for patients with initial consultations from January to June 2019 (pre-fibroid center) and from January to June 2021 (post-fibroid center). **Results**: Among 615 patients meeting inclusion criteria, 273 had consultations pre-center and 342 post-center. More patients seen post-center had previously attempted medical management (30.1% vs. 20.2%), with a significant proportion having no prior medical or surgical treatment (53.2% vs. 61.5%). Post-center, there were more MIGS consultations (65.5% vs. 53.1%) and a decrease in general gynecology (GYN) consultations (19.0% vs. 25.6%). More patients sought additional opinions post-center (83.6% vs. 67.0%), particularly with MIGS (58.8% vs. 37.0%). General GYNs referred to MIGS (79.3% vs. 73.1%) and IR specialists (16.0% vs. 13.0%) more often in 2021. In 2021, use of MRI increased (66.5% vs. 52.4%), and more patients underwent uterine artery embolization (UAE) within 1 year of consultation compared to the pre-center period (13.8% vs. 6.9%). **Conclusions**: Patients with symptomatic fibroids often seek the expertise of specialists to explore treatment options. A multidisciplinary fibroid center that integrates efforts of MIGS and IR enables thorough counseling and a rise in the utilization of minimally invasive procedures, including UAE.

## 1. Introduction

When multiple specialties are involved in the care of the same medical condition, there may be competition for patient volume and revenue rather than collaboration. Moreover, gynecologists and interventional radiologists exhibit different specialty-specific values and perspectives regarding their treatment of uterine fibroids [1]. Fibroid symptoms such as heavy menstrual bleeding, pelvic pain, and bulk-related symptoms may be managed by gynecologists medically via hormonal and non-hormonal medications or surgically via hysterectomy or myomectomy, for those desiring fertility or uterine-sparing procedures [2,3,4,5,6]. In the 1990s, uterine artery embolization (UAE), performed by interventional radiologists, emerged as an effective, minimally invasive nonsurgical fibroid treatment [7,8,9].

There are no established guidelines on how patients should be optimally referred for surgical versus interventional procedures. Historically, some gynecologists have been hesitant to refer eligible patients to an interventional radiologist for UAE due to fibroid size, location, and pregnancy concerns [10,11]. This has led to increased numbers of self-referrals among patients for UAE due to more awareness of the procedure, and many patients who undergo UAE are not up to date on their routine gynecological care [12]. Multidisciplinary collaboration optimizes patient outcomes, increases provider satisfaction, and improves cost-effectiveness [13,14]. Gynecologists and interventional radiologists who have established referral relationships and can comprehensively counsel patients on their wide array of treatment options may help advance patient care.

Several multidisciplinary fibroid programs have demonstrated high patient satisfaction while also increasing use of minimally invasive treatment options [15,16,17,18,19]; however, their effects on changes in patient referral patterns and use of imaging modalities remain relatively unknown. To assess how collaboration affects patient care and impacts fibroid treatment, referral patterns and management of symptomatic fibroids were examined before and after implementation of a multidisciplinary fibroid center with minimally invasive gynecologic surgery (MIGS) and interventional radiology (IR). The aim of this study was to contribute to the limited literature on formal centers of collaboration between these specialties for fibroid care.

## 2. Materials and Methods

This retrospective cohort study was conducted at a single urban academic medical center. In September 2020, a multidisciplinary fibroid center was established in which patients referred to the center are offered same-day consultations with minimally invasive gynecologic surgeons (MIGSs) and with interventional radiologists (IRs) within three weeks of appointment request. MIGSs and IRs were chosen to participate in the fibroid center, as they are the primary groups treating fibroids at our institution and can offer comprehensive minimally invasive treatment options. The fibroid center was open one and a half days per week, with a rotating schedule of MIGS and IR providers. MIGS and IR appointments were conducted separately. IR visits were conducted mostly via telemedicine for ease of scheduling, since IR had already transitioned to primary video visits following the COVID-19 pandemic. In-person appointments were an option if the patient requested them or if deemed necessary by the IR provider. MIGS consultations were usually performed in the office prior to IR appointments to allow for pelvic exams, rule out other gynecologic pathology, and perform endometrial biopsy when indicated, though occasionally, IRs would see patients first if scheduling issues arose. If patients were referred directly to the IR for fibroids and UAE, the patients were also referred to be seen by MIGSs within the fibroid center. Patients were able to decline consultation with either MIGSs or IRs if desired. The scheduling process was streamlined so that any patient calling the department scheduling number with a complaint of fibroids was referred directly to the fibroid center, as opposed to before the fibroid center, when patients were scheduled with any available private gynecology provider. Surgical fibroid management options offered by MIGSs pre- and post-center included hysteroscopic myomectomy, minimally invasive and abdominal myomectomy and hysterectomy, and laparoscopic radiofrequency ablation. Minimally invasive surgical approaches included traditional laparoscopic myomectomy and hysterectomy, robot-assisted laparoscopic myomectomy and hysterectomy, and vaginal hysterectomy and were offered based on anatomy, surgical complexity, and surgeon preference. Post-center, options expanded to include office diagnostic hysteroscopy and transcervical radiofrequency ablation. Fibroid treatment options offered by IRs included UAE and MR-guided focused ultrasound (MRgFUS); however, MRgFUS was not frequently chosen by patients following evidence-based counseling. No additional staff were hired upon creation of the fibroid center, though existing staff and patient care spaces were specifically designated for use by the fibroid center. Follow-up appointments after initial consultation were scheduled at the provider’s discretion as needed, and there were no systematic changes to the number or timing of follow-up appointments post-center compared to pre-center. 

Following these consultations, monthly interdisciplinary meetings were held between providers regarding select cases to ensure ongoing discussion of complex cases and comprehensive fibroid management. Each month, approximately 5–10 challenging cases were submitted to an administrator to distribute to the fibroid center faculty. Examples of such cases included markedly enlarged fibroids, difficult fibroid location (ex. cervical or intracavitary fibroid), underlying adenomyosis, atypical-appearing fibroid on magnetic resonance imaging (MRI), or other complex cases at the provider’s discretion. The patient’s history, imaging, and tentative plan were reviewed during the meetings. All cases discussed were intended for a collaborative decision and group consensus regarding the most effective treatment options tailored to the patients’ individual needs. These meetings at times changed or modified the initial treatment plan. Providers communicated the group consensus regarding their best treatment option/s with their patients thereafter, who were able to ask questions and address concerns regarding management recommendations with either their MIGS or IR provider. Collaboration also occurred between MIGSs and IRs outside of these scheduled meetings to facilitate discussion with the patients around and during their office visits.

Prior to the fibroid center, patients were referred on an individual basis to either the MIGS or the IR at the discretion of the referring provider or were self-referred; this was also the case post-center. However, self-referrals were previously evaluated by their gynecologists and given a fibroid diagnosis. The fibroid center was advertised via marketing by the department through announcements and presentations at all affiliated hospital sites, as well as through hospital media. Many referrals to the fibroid center came from the reproductive endocrinologists (REIs) at the institution. Notably, REIs in the obstetrics and gynecology department were not fibroid center consultants, as they do not primarily treat fibroids or offer laparoscopic or robotic myomectomy. Patient care plans were routinely discussed, and referrals were made to REI providers if infertility was a concern. 

All patients who presented for initial fibroid consultation between January and June 2019 and January and June 2021 were included in the study. These date ranges were chosen to examine differences in demographics, fibroid characteristics, referral patterns, and treatments before and after establishment of the fibroid center. Due to new establishment of the fibroid center and back-up of cases from the COVID-19 pandemic in 2020, the 2021 time period was chosen, as this was when patient and surgical volume had returned to pre-pandemic levels. Providers in 2019 were not aware that a fibroid center would be established in 2020. Inclusion criteria were genetically female patients aged 18–99 who had uterine fibroids on ultrasound or MRI. Consultation providers included general gynecologists (GYNs), MIGSs, IRs, and others (mainly including gynecologic subspecialties such as reproductive endocrinology, urogynecology, and gynecologic oncology). Of these providers, four full-time MIGSs, three IR physicians, and two IR nurse practitioners were consultants in the fibroid center, and these 36 same-day fibroid center consultations were included in the 2021 post-center group. Post-center, some patients saw a general GYN or other specialist rather than fibroid center providers, as they may not have had a clear diagnosis of fibroids or concern for fibroids when making their visit, but needed an annual GYN examination and were found to have fibroids, or they were specifically referred to see a provider not in the fibroid center. Patient charts to review for possible inclusion in the study were selected from primary and secondary International Classification of Diseases (ICD) and Current Procedural Terminology (CPT^®^) codes. 

This study was approved by the Institutional Review Board. Patient demographics, clinical characteristics, medical history, referral patterns, and management after consultation were retrieved from the electronic medical record. Baseline imaging was defined as any radiologic study that was obtained prior to and available at the time of their initial appointment, as well as imaging ordered by a MIGS or IR provider at the time of consultation. Almost all patients presented with some type of baseline imaging, and patients were recommended to send any imaging obtained within the prior year, but this was not required to make an appointment with the fibroid center. Saline infusion ultrasound (SIS) was ordered as needed at the discretion of the provider to evaluate fibroids with submucosal components (FIGO types 0–2). Fibroid characteristics were determined based on available images, with preference to MRI results over ultrasound when both were available, as MRI provides more detail regarding number, size, and location of fibroids and helps guide mapping of fibroids prior to surgery [20,21]. Patients considering UAE or myomectomy were recommended to obtain an MRI to better guide treatment. The data were collected via manual chart review by a single medical provider with oversight by two additional providers to ensure consistency. Study data were anonymously managed and analyzed using REDCap (version 12.4.0) (Research Electronic Data Capture) electronic data capture tools.

Descriptive statistics were reported as frequencies (percentages) for categorical variables and as means (standard deviations) for continuous variables. A chi-square test or Fisher’s exact test was performed, as appropriate, to compare the difference in proportions between 2019 pre-center and 2021 post-center patients. Two-sample *t* tests and Wilcoxon rank sum tests were performed to compare the difference in the mean value of the continuous measures. All *p*-values were two-sided, with statistical significance evaluated at the 0.05 alpha level. Data analyses were performed using R Statistical Software (v4.1.2; R Core Team 2021).

## 3. Results

A total of 615 initial fibroid consultations were performed during the study period: 273 consults in January–June 2019 (pre-center) and 342 consults in January–June 2021 (post-center). The groups were similar in most baseline characteristics (Table 1). The mean patient age was 44.3 years pre-center vs. 44.4 years post-center (*p* = 0.915), mostly pre-menopausal, with a median BMI of 26.6 vs. 25.8 (*p* = 0.159). Patients were racially diverse, with 33.0% pre-center vs. 32.5% post-center identifying as white, 32.2% vs. 26.6% as black, 21.6% vs. 23.4% as another race, and 5.9% vs. 10.5% as Asian (*p* = 0.214). In addition, 11.4% vs. 8.2%% of patients identified as Hispanic ethnicity (*p* = 0.185). With respect to any prior abdominal surgery, 25.3% vs. 30.4% (*p* = 0.159) underwent laparotomy and 20.5% vs. 20.5% (*p* = 0.989) underwent laparoscopy. Patients seen pre-center were less likely to have private insurance (80.6% vs. 92.4%, *p* < 0.001) than the 2021 post-center group.

Regarding fibroid characteristics on ultrasound or MRI prior to consultation or ordered at the initial appointments by either the MIGS or IR, median total fibroid number (three fibroids pre-center vs. three post-center, *p* = 0.99), mean maximum diameter of largest fibroid (6.9 cm vs. 7.7 cm, *p* = 0.37), and mean uterine volume (605.2 cm^3^ vs. 653.3 cm^3^, *p* = 0.18) were similar between the pre- and post-center groups (Table 2). Fewer intramural fibroids were noted upon imaging in the pre-center group (81.8% vs. 87.8%, *p* = 0.039), but otherwise, fibroid types were comparable between the two time periods, including submucosal (FIGO types 0–2) (38.8% vs. 34.5%, *p* = 0.277), subserosal (FIGO types 5–6) (60.6% vs. 62.2%, *p* = 0.686), and pedunculated (FIGO type 7) (18.2% vs. 15.2%, *p* = 0.326). Some patients had radiographic evidence of other gynecologic pathologies such as endometriosis (6.3% vs. 6.8%, *p* = 0.804) and adenomyosis (10.0% vs. 11.3%, *p* = 0.624), and these did not vary between the time periods.

Table 3 is a summary of patient clinical information related to their fibroids. The most common presenting symptoms included bleeding (68.1% pre-center vs. 70.2% post-center, *p* = 0.585), bulk (49.8% vs. 53.5%, *p* = 0.363), and pain (39.2% vs. 44.7%, *p* = 0.167). No symptoms were reported by 10.3% vs. 6.1% of patients (*p* = 0.061). Most patients had an ultrasound prior to or ordered at their consultation (68.5% vs. 71.9%, *p* = 0.354). Notably, the number of magnetic resonance imaging (MRI) scans performed increased over the study period, from 137 (50.2%) pre-center to 207 (60.53%) post-center (*p* = 0.01). The increased utilization of MRI imaging post-center among MIGSs (52.4% pre-center vs. 66.5% vs. post-center, *p* < 0.01) accounted for this difference. Patients seen pre-center were less likely to have previously tried medical management than patients seen post-center (20.2% vs. 30.1%, *p* < 0.01), including hormonal birth control, nonsteroidal anti-inflammatory drugs (NSAIDs), tranexamic acid, intrauterine devices, and GnRH agonist or antagonists, but the majority had no prior treatment (61.5% vs. 53.2%, *p* = 0.04). Specifically looking at prior surgical management of fibroids, 9.2% vs. 9.4% (*p* = 0.933) of patients had undergone abdominal myomectomy, and 2.9% vs. 4.1% (*p* = 0.440) had history of laparoscopic or robotic myomectomy. More patients presented for an additional opinion after establishment of the fibroid center (67.0% pre-center vs. 83.6% post-center, *p* < 0.01), predominately with MIGSs (37.0% pre-center vs. 58.8% post-center, *p* < 0.01).

**Figure 1:**
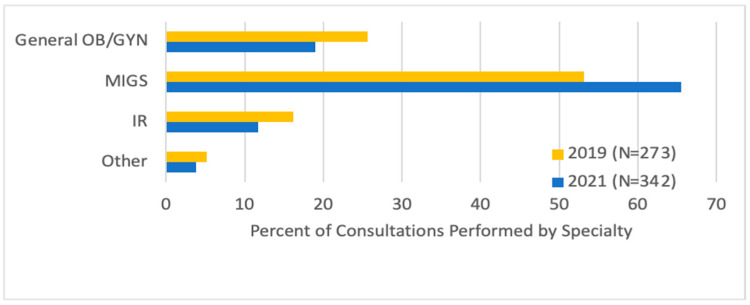
Providers by specialty who performed initial fibroid consultation pre-center (2019) versus post-center (2021) (*p* = 0.021).

**Figure 2:**
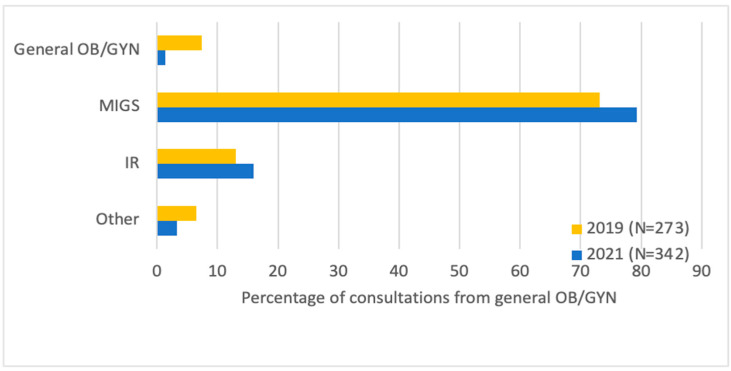
Specialties referred to by general OB/GYN pre-center (2019) versus post-center (2021) (*p* = 0.046).

**Figure 3:**
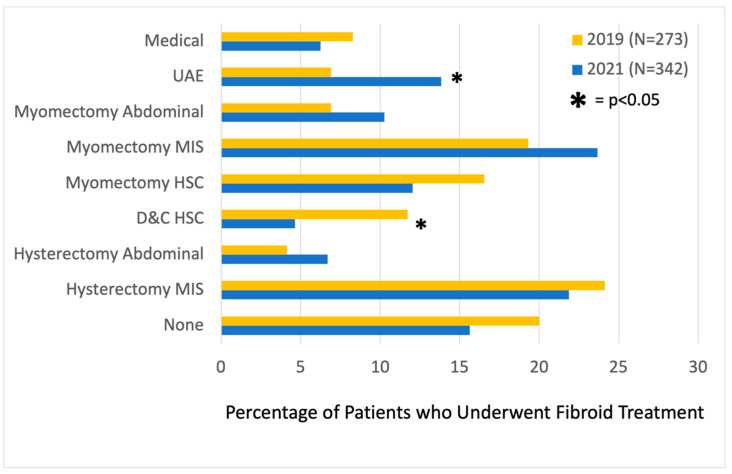
Fibroid management within 1 year of initial consultation by MIGS.

Looking at referrals, there were more MIGS (65.5% vs. 53.1%) and fewer general GYN (19.0% vs. 25.6%, *p* = 0.02) initial consultations after the fibroid center was established, and fewer patients (16.1% vs. 11.7%, *p* = 0.02) underwent consultation directly with an IR (Figure 1). Most patients were either referred by their general GYN (42.0%) or were self-referred (41.3%). General GYNs were more likely to refer to MIGSs (79.3% vs. 73.1%) and IRs (16.0% vs. 13.0%, *p* = 0.046) post-center, and this accounted for the majority of the difference in number of consultations (Figure 2). Among patients who saw an MIGS for consultation post-center, more underwent uterine artery embolization (UAE) (13.8% vs. 6.9%, *p* = 0.04). In terms of procedures performed, fewer underwent diagnostic hysteroscopy/dilation and curettage (11.7% vs. 4.5%, *p* < 0.01) within 1 year of consultation. There was no statistically significant difference in the number of patients post-center compared to pre-center who proceeded with medical management (6.3% vs. 8.3%, *p* = 0.458), abdominal myomectomy (10.3% vs. 6.9%, *p* = 0.268), minimally invasive myomectomy (23.7% vs. 19.3%, *p* = 0.324), hysteroscopic myomectomy (12.1% vs. 16.6%, *p* = 0.221), open hysterectomy (6.7% vs. 4.1%, *p* = 0.300), minimally invasive hysterectomy (21.9% vs. 24.1%, *p* = 0.613), or no treatment (15.6% vs. 20.0%, *p* = 0.278) (Figure 3).

## 4. Discussion

The findings of this retrospective cohort study demonstrate that introducing a multidisciplinary fibroid center with collaborative and directed management by MIGS and IR leads to comprehensive counseling and an increase in minimally invasive procedures, including UAE. After implementation of the fibroid center, both MIGS and IR consultations increased, indicating that establishing a multidisciplinary fibroid center can increase referral volumes for both specialties and be mutually beneficial. 

To examine changes in referral patterns and fibroid management before and after the fibroid center was established in September 2020, patient groups were compared in six-month intervals from January to June 2019 (pre-center) and from January to June 2021 (post-center). Almost all patients who presented for initial consultation (92%) exhibited symptoms from their fibroids, including bleeding, pain, bulk, or infertility. After the center was established, the number of consultations by MIGS specialists significantly increased from 53% to 65%, and many of these MIGS specialists referred to IRs either within the organization of the fibroid center (36 same-day consultations with MIGSs and IRs) or separately. Referrals from general OB/GYNs to the fibroid center accounted for most of this difference. Compared to pre-center, patients seen post-center were more likely to have previously attempted other treatments for their fibroids and were seeking additional specialist opinions, demonstrating a need in the community for specialist care of symptomatic fibroids. Through the center, there was a notable increase in patients undergoing minimally invasive uterine-sparing procedures, including UAE, and a decrease in less effective diagnostic and therapeutic dilation and curettage (D&C) procedures. Fewer patients saw an IR as their first point of contact post-center, the implications of which may warrant further investigation as to whether they were less likely to delay general gynecologic care or more likely to pursue workup of alternative causes of abnormal uterine bleeding and endometrial sampling.

Competition for patient volume can improve healthcare quality but may incentivize physicians to engage in opportunistic behaviors [22]. These behaviors may depend on the weight the physician places on profit, but they can be affected by altruistic motivations to help their patients or social motivations in collaborative settings [23]. Potential for care bias based on specialty-specific knowledge, personal values, or financial reimbursement may result in suboptimal management and affect patient outcomes [22,23,24]. When multiple specialties are involved in treating the same condition, such as gynecologists and interventional radiologists with regard to symptomatic fibroids, this is especially apparent [13]. While both gynecologists and interventional radiologists educate their patients about UAE, interventional radiologists are more likely to recommend UAE than their gynecologist counterparts [25]. UAE and myomectomy were recently compared in the Fibroids with Either Embolization of Myomectomy to Measure the Effect on Quality of Life (FEMME) trial, which concluded that patients who underwent myomectomy had a better fibroid-related quality of life at 2 years than UAE [26]. However, these findings have been met with some criticism by interventional radiologists, in that UAE was cast in an unfavorable light and UAE may be underutilized [27,28]. 

Previous work shows that collaboration between specialties actually improves patient outcomes and decreases cost [13,14]. Gynecologists and interventional radiologists exhibit different and wide-ranging perspectives on optimal treatment of symptomatic fibroids. When working together, they can best counsel patients on their full range of treatment options [1]. Several multidisciplinary fibroid programs have demonstrated an expansion of minimally invasive procedures while also increasing patient and provider satisfaction [15,16,17]. The desire to elevate the patient care experience and advance minimally invasive fibroid treatments led our institution to establish a multidisciplinary fibroid center staffed by MIGSs and IRs. 

Compared to other multidisciplinary fibroid clinics [15,16,17], this center is unique in that all patients are offered appointments with both an MIGS and an IR on the same day. This allows patients to be adequately counseled on all minimally invasive medical, surgical, and interventional treatment options by the same providers that perform those procedures, helping to better educate patients to make their own informed care decisions that are most in line with their goals of care. Without this aspect of the fibroid center, it is unlikely that patients would be referred for or self-schedule the two separate appointments [29]. Additionally, the center can schedule patients for consultations within three weeks of appointment request, allowing for prompt care and increased patient satisfaction. A survey study performed at this fibroid center confirms that many patients with symptomatic fibroids are not being adequately counseled about their management options, and nearly all (95%) were more knowledgeable about their treatment options after their care at the center [29]. Interestingly, the number of initial IR consultations did not change significantly between the study periods, yet the amount of UAE procedures being performed increased compared to other fibroid treatments. It may be hypothesized that the center facilitated a more neutral setting through which competition played less of a factor compared to patient education and shared decision-making. Indeed, the interdisciplinary monthly meetings encourage shared decision-making, facilitate ongoing discussion of complex cases, and foster a collaborative environment with a new shared identity through comradery. There was also an increase in the use of MRI as the primary imaging modality post-center, which may have improved accuracy of fibroid diagnosis and aided in pre-procedure planning and guiding treatments. It may be correlated to the increase in UAE procedures, as typically, all patients undergoing UAE at the fibroid center get an MRI.

Strengths of this study include its examination of a unique model for comprehensive multidisciplinary fibroid programs. The data also add to the paucity of literature studies regarding changes in practice patterns and management types after implementation of fibroid programs. The study’s limitations include its analysis of a single academic medical system, which limits its external validity and may exhibit less financial motivation than a private practice model. The implementation of a comprehensive multidisciplinary fibroid center also involves offering many types of treatment, and the results cannot be generalized, as many institutions and countries may have limited access to certain treatments. The patient population also included a high number of privately insured patients as opposed to patients enrolled in public health programs, although Medicaid patients have access to MIGS and IR specialists. The number of physicians involved in the center during the study period included four MIGSs and three IRs, and patient management from this small number may not be reflective of other providers in these specialties. Laparoscopic radiofrequency ablation (RFA) was only offered by one MIGS who performed the procedure; however, no patients elected to undergo RFA during the study period. Patients who requested transcervical RFA during the study period were referred to institutions that provided this treatment, although this technology has now been acquired and is offered as an option at the fibroid center. MR-guided focused ultrasound (MRgFUS) is an alternative uterine-sparing treatment offered at the fibroid center; however, no patients chose this as their treatment during the study period. As with all retrospective studies, the study participants were recruited by convenience sampling and are prone to selection bias. Data collection via chart review is prone to recall or misclassification bias, although variance was mitigated by use of a single medical provider extracting information from the electronic medical record. Lastly, factors involved in changes to physician practice patterns are often complex, and all contributing variables may not have been captured by the study. Patient and provider satisfaction, cost analysis, and long-term patient outcomes were also not investigated and could be an important consideration for future analysis. A prospective study comparing the practice patterns, outcomes, and patient satisfaction of the multidisciplinary fibroid center to the traditional model, where patients with fibroids consult with providers in separate MIGS and IR departments, could also be considered as a point of future investigation.

In summary, the implementation of a collaborative, multidisciplinary fibroid center with MIGS and IR consult providers led to higher patient volume, especially for additional specialist opinions, and increased the number of uterine-sparing and minimally invasive procedures performed, including UAE. A coordinated effort between physician specialties provides comprehensive, timely evaluation. The impact of the results of this study may contribute to the field of fibroid management and potentially guide the development of multidisciplinary fibroid centers in other healthcare settings.

## Figures and Tables

**Table 1:** Patient demographics and surgical history for those who had consultation for fibroids.

	2019 (*n* = 273)	2021 (*n* = 342)	*p* Value
Age, Mean (SD)	44.3 (9.5)	44.4 (9.4)	0.915
Insurance Type, N (%)			<0.001
Private	220 (80.6)	316 (92.4)	
Medicare	13 (4.8)	9 (2.6)	
Medicaid	40 (14.7)	17 (5.0)	
Gravidity, Median (IQR)	1 (0, 2)	1 (0, 2)	0.029
Nulliparous, N (%)	152 (55.7)	195 (57.0)	0.739
BMI, Median (IQR)	26.6 (23.6, 30.7)	25.8 (22.8, 30.2)	0.159
Race, N (%)			0.214
White	90 (33.0)	111 (32.5)	
Black	88 (32.2)	91 (26.6)	
Asian	16 (5.9)	36 (10.5)	
Other	59 (21.6)	80 (23.4)	
Multiple race	20 (7.3)	24 (7.0)	
Ethnicity, N (%)			0.185
Hispanic	31 (11.4)	28 (8.2)	
Non-Hispanic	242 (88.6)	314 (91.8)	
Menopausal Status, N (%)			0.147
Premenopausal	243 (89.0)	316 (92.4)	
Postmenopausal	30 (11.0)	26 (7.6)	
History of Laparotomy, N (%)			0.159
Yes	69 (25.3)	104 (30.4)	
No	204 (74.7)	238 (69.6)	
History of Laparoscopy, N (%)			0.989
Yes	56 (20.5)	70 (20.5)	
No	217 (79.5)	272 (79.5)	

**Table 2:** Fibroid imaging characteristics.

	2019 (*n* = 273)	2021 (*n* = 342)	*p* Value
Total fibroid number, median (IQR)	3 (2, 5)	3 (2, 4)	0.992
Average max diameter of largest fibroid (cm), mean (SD)	6.9 (3.9)	7.7 (10.7)	0.377
Average uterine volume (cm^3^), mean (SD)	605.2 (635.4)	653.3 (711.1)	0.184
Fibroid types, N (%)			
Submucosal	104 (38.8)	116 (34.5)	0.277
FIGO Type 0	25 (24.3)	19 (17.1)	
FIGO Type 1	8 (7.8)	16 (14.4)	
FIGO Type 2	32 (31.1)	22 (19.8)	
Not available	38 (36.9)	54 (48.6)	
Intramural (FIGO Types 3–4)	220 (81.8)	295 (87.8)	0.039
Subserosal (FIGO Types 5–6)	163 (60.6)	209 (62.2)	0.686
Pedunculated (FIGO Type 7)	49 (18.2)	51 (15.2)	0.326
Endometriosis noted on imaging, N (%)	17 (6.3)	23 (6.8)	0.804
Adenomyosis noted on imaging, N (%)	27 (10.0)	38 (11.3)	0.624

**Table 3:** Clinical information.

	2019 (*n* = 273)	2021 (*n* = 342)	*p* Value
Symptom profile, N (%)	186 (68.1)	240 (70.2)	0.585
Abnormal uterine bleeding	136 (49.8)	183 (53.5)	0.363
Bulk	21 (7.7)	15 (4.4)	0.083
Infertility	107 (39.2)	153 (44.7)	0.167
Pain	28 (10.3)	21 (6.1)	0.061
Asymptomatic	1 (0.4)	4 (1.2)	0.389
Other			
Baseline imaging type, N (%)	187 (68.5)	246 (71.9)	0.354
Pelvic ultrasound	137 (50.2)	207 (60.5)	0.010
Magnetic resonance imaging (MRI)	6 (2.2)	2 (0.6)	0.148
Other	4 (1.5)	4 (1.2)	0.738
None			
Prior fibroid management, N (%)	55 (20.2)	103 (30.1)	0.005
Medical Management	12 (4.4)	17 (5.0)	0.738
Uterine artery embolization (UAE)	25 (9.2)	32 (9.4)	0.933
Myomectomy abdominal	8 (2.9)	14 (4.1)	0.440
Myomectomy—laparoscopy	14 (5.1)	22 (6.4)	0.494
Myomectomy—hysteroscopy	12 (4.4)	8 (2.3)	0.153
Hysteroscopy dilation and curettage (D&C)	2 (0.7)	4 (1.2)	0.698
Endometrial Ablation	168 (61.5)	182 (53.2)	0.038
None	183 (67.0)	286 (83.6)	<0.001
Prior eval by general OBGYN, N (%)	186 (68.1)	240 (70.2)	0.585

## Data Availability

The data that support the findings of this study are not openly available due to reasons of sensitivity and are available from the corresponding author upon reasonable request. Data are located in controlled access data storage via RedCap at Weill Cornell Medicine.
